# Influenza vaccine effectiveness in Europe: Results from the 2022–2023 VEBIS (Vaccine Effectiveness, Burden and Impact Studies) primary care multicentre study

**DOI:** 10.1111/irv.13243

**Published:** 2024-01-10

**Authors:** Marine Maurel, Francisco Pozo, Gloria Pérez‐Gimeno, Silke Buda, Noémie Sève, Beatrix Oroszi, Mariette Hooiveld, Verónica Gomez, Lisa Domegan, Iván Martínez‐Baz, Maja Ilić, Anna Sara Carnahan, Maria Elena Mihai, Ana Martínez, Luise Goerlitz, Vincent Enouf, Judit Krisztina Horváth, Frederika Dijkstra, Ana Paula Rodrigues, Charlene Bennett, Camino Trobajo‐Sanmartín, Ivan Mlinarić, Neus Latorre‐Margalef, Alina Ivanciuc, Aurora Lopez, Ralf Dürrwald, Alessandra Falchi, Gergő Túri, Adam Meijer, Aryse Melo, Joan O'Donnell, Jesús Castilla, Vesna Višekruna Vučina, Tove Samuelsson Hagey, Mihaela Lazar, Marlena Kaczmarek, Sabrina Bacci, Esther Kissling, VEBIS study team

**Affiliations:** ^1^ Epiconcept Paris France; ^2^ National Centre for Microbiology Institute of Health Carlos III Madrid Spain; ^3^ Consortium for Biomedical Research in Epidemiology and Public Health (CIBERESP) Madrid Spain; ^4^ Department for Infectious Disease Epidemiology, Respiratory Infections Unit Robert Koch Institute Berlin Germany; ^5^ Sorbonne Université INSERM, Institut Pierre Louis d'épidémiologie et de Santé Publique (IPLESP UMRS 1136) Paris France; ^6^ National Laboratory for Health Security, Epidemiology and Surveillance Centre Semmelweis University Budapest Hungary; ^7^ Nivel Utrecht the Netherlands; ^8^ Instituto Nacional de Saúde Dr. Ricardo Jorge Lisbon Portugal; ^9^ HSE‐Health Protection Surveillance Centre Dublin Ireland; ^10^ Instituto de Salud Pública de Navarra (IdiSNA) Pamplona Spain; ^11^ Croatian Institute of Public Health Zagreb Croatia; ^12^ The Public Health Agency of Sweden (PHAS) Stockholm Sweden; ^13^ “Cantacuzino” National Military Medical Institute for Research and Development Bucharest Romania; ^14^ Subdirección General de Vigilancia y Respuesta a Emergencias de Salud Pública, Agencia de Salud Pública de Catalunya Barcelona Spain; ^15^ Centre National de Référence Virus des Infections Respiratoire (CNR VIR), Institut Pasteur Paris France; ^16^ National Institute for Public Health and the Environment (RIVM) Bilthoven the Netherlands; ^17^ National Virus Reference Laboratory University College Dublin Dublin Ireland; ^18^ Subdirección General de Epidemiologia y Vigilancia de la Salud Valencia Spain; ^19^ National Reference Centre for Influenza Robert Koch Institute Berlin Germany; ^20^ Laboratoire de Virologie Université de Corse‐Inserm Corte France; ^21^ European Centre for Disease Prevention and Control Stockholm Sweden

**Keywords:** Europe, influenza, influenza vaccine, multicentre study, vaccine effectiveness

## Abstract

**Background:**

Influenza A(H3N2) viruses dominated early in the 2022–2023 influenza season in Europe, followed by higher circulation of influenza A(H1N1)pdm09 and B viruses. The VEBIS primary care network estimated the influenza vaccine effectiveness (VE) using a multicentre test‐negative study.

**Materials and Methods:**

Primary care practitioners collected information and specimens from patients consulting with acute respiratory infection. We measured VE against any influenza, influenza (sub)type and clade, by age group, by influenza vaccine target group and by time since vaccination, using logistic regression.

**Results:**

We included 38 058 patients, of which 3786 were influenza A(H3N2), 1548 influenza A(H1N1)pdm09 and 3275 influenza B cases. Against influenza A(H3N2), VE was 36% (95% CI: 25–45) among all ages and ranged between 30% and 52% by age group and target group. VE against influenza A(H3N2) clade 2b was 38% (95% CI: 25–49). Overall, VE against influenza A(H1N1)pdm09 was 46% (95% CI: 35–56) and ranged between 29% and 59% by age group and target group. VE against influenza A(H1N1)pdm09 clade 5a.2a was 56% (95% CI: 46–65) and 79% (95% CI: 64–88) against clade 5a.2a.1. VE against influenza B was 76% (95% CI: 70–81); overall, 84%, 72% and 71% were among 0–14‐year‐olds, 15–64‐year‐olds and those in the influenza vaccination target group, respectively. VE against influenza B with a position 197 mutation of the hemagglutinin (HA) gene was 79% (95% CI: 73–85) and 90% (95% CI: 85–94) without this mutation

**Conclusion:**

The 2022–2023 end‐of‐season results from the VEBIS network at primary care level showed high VE among children and against influenza B, with lower VE against influenza A(H1N1)pdm09 and A(H3N2).

## 1 INTRODUCTION

Influenza viruses undergo rapid evolution, leading to regular reformulations of the influenza strains in the influenza vaccines. The World Health Organisation (WHO) recommended that the 2022–2023 trivalent egg‐based influenza vaccine for the Northern Hemisphere includes an A/Victoria/2570/2019 (H1N1)pdm09‐like virus, an A/Darwin/9/2021 (H3N2)‐like virus and a B/Austria/1359417/2021 (B/Victoria lineage)‐like virus. It was recommended that quadrivalent vaccines containing two influenza B viruses should contain the three viruses mentioned above as well as a B/Phuket/3073/2013 (B/Yamagata lineage)‐like virus.^1^

In the 2022–2023 influenza season in the WHO European region, there was an initial dominance of influenza A(H3N2) circulation, followed by higher circulation of influenza A(H1N1)pdm09 and B viruses.^2^ The circulation of influenza A(H3N2), A(H1N1)pdm09 and B viruses varied by country.^2^

Since July 2022, the I‐MOVE (Influenza Monitoring Vaccine Effectiveness in Europe) network has become integrated into the ECDC (European Centre for Disease Prevention and Control) VEBIS (Vaccine Effectiveness, Burden and Impact Studies) project. The network has been estimating influenza vaccine effectiveness (VE) at primary care level in the European Union (EU) and European Economic Area (EEA) since 2008–2009, using a multicentre study design including up to 12 countries.^3^, ^4^, ^5^, ^6^, ^7^, ^8^ In countries participating in the network, influenza vaccination is recommended among older adults aged ≥50, ≥60 or ≥65 years (depending on country), those within medical risk groups for severe disease and among children (aged 2–17 years) in Ireland and Spain.^9^, ^10^

We report the 2022–2023 seasonal VE against influenza by (sub)type and clade‐specific, overall, by age group and by influenza vaccination target group among patients presenting to participating primary care sites in Europe with an acute respiratory infection.

## 2 METHODS

Eleven European study sites from 10 countries (located in Croatia, France, Germany, Hungary, Ireland, the Netherlands, Portugal, Romania, Spain national, Spain Navarra region and Sweden) participated in the 2022–2023 VEBIS multicentre test‐negative case–control study. The methods are based on the ECDC generic case–control study protocol and the I‐MOVE+ generic study protocol.^4^, ^11^, ^12^, ^13^

Participating practitioners collected specimens and interviewed all or a systematic sample of patients consulting for acute respiratory infection (ARI) or influenza‐like illness (ILI). The common variables collected in all study sites were symptoms, date of onset, date of specimen collection, 2022–2023 seasonal influenza vaccination status and date, sex, age and presence of chronic conditions. All specimens were PCR‐tested for influenza virus, as well as for SARS‐CoV‐2.

In a pooled analysis, we included patients with a specimen taken less than 8 days after symptom onset and meeting the EU ARI or ILI case definition. Patients testing PCR‐positive for influenza virus were designated as cases and those testing negative for any influenza virus as controls.

For each study site, we included patients presenting with symptoms 14 or more days after the start of national influenza vaccination campaigns. Controls were excluded if they presented prior to the onset week of the first influenza (sub)type positive case for each (sub)type‐specific analysis.

A patient was considered as vaccinated if he had received at least one dose of 2022–2023 influenza vaccine 14 or more days before symptom onset. Patients vaccinated fewer than 14 days before symptom onset were excluded. All the others were classified as unvaccinated. Influenza vaccination status was ascertained from electronic medical records by practitioners, obtained through linkage to national vaccine registries or was self‐reported.

In one study site, where date of symptom onset was not available, we imputed it as 2 days before the swab date, as 2 days was the median delay between onset and swab in the pooled data.

We excluded any study site from the pooled analysis that had fewer than 10 influenza‐type‐specific cases or controls. We combined individual patient data and used a one‐stage model, with study site as a fixed effect. We estimated influenza VE as (1 − [odds of influenza vaccination among cases/odds of influenza vaccination among controls]) × 100. We conducted a complete case analysis and used logistic regression to estimate VE, including a priori potential confounding factors: age, sex, presence of at least one commonly collected chronic condition (including lung disease, heart disease, immunodeficiency and diabetes) and date of symptom onset. For continuous variables, we used age categorised in narrow groups (0–1, 2, 3–4, 5–9, 10–19, 20–29, …, 60–69, 70+ years), as a linear term or as a restricted cubic spline (with three, four or five knots) and symptom onset date as a restricted cubic spline (with three, four or five knots). We used the Akaike information criterion (AIC) to select the best functional form of the continuous variables.

We estimated VE for any influenza and by influenza (sub)type and, where sample size allowed, stratified by age group (0–14, 15–64, 65 years and older) and by target group for influenza vaccination. We estimated VE by time since vaccination, using intervals of 14–89, 90–119 and ≥120 days for influenza A(H1N1)pdm09 and influenza B. For influenza A(H3N2), because of the shorter season and earlier presentation, we used 14–59, 60–89 and ≥90 days intervals.

We conducted a sensitivity analysis excluding SARS‐CoV‐2‐positive controls to account for the correlation between influenza and COVID‐19 vaccination that could bias VE estimates.^14^

If the number of cases or controls were fewer than 10 times the number of parameters in a model, we used penalised logistic regression (Firth's method) to assess small sample bias.^15^ If the VE differed by 10% or more, we assumed there was a small sample bias and did not present the results.

Nine of 11 study sites participated in the laboratory sequencing protocol, where all or a random sample of influenza viruses were selected for sequencing (mainly the whole haemagglutinin (HA) segment but also the HA1 subunit). In one study site (France), only samples going to one of three laboratories used within the study carried out sequencing. Both the whole HA and the HA1 subunit sequences were uploaded by each site to GISAID and downloaded for centralised phylogenetic and amino acid substitution analysis in MEGA7 to determine clade distribution at the National Influenza Centre, Madrid. For the clade‐specific VE analysis, we excluded any study site that had fewer than five cases of that influenza clade.

As not all study sites attempted to sequence 100% of viruses across the season, we carried out an analysis taking the sampling fraction into account. The logistic regression model was weighted using the reciprocal of the fraction of samples sequenced (sequencing fraction) within each period with different sampling fractions and robust standard errors were used. The sequencing fraction was defined for each study site for each (sub)type for different periods within the season.

## 3 RESULTS

**FIGURE 1:**
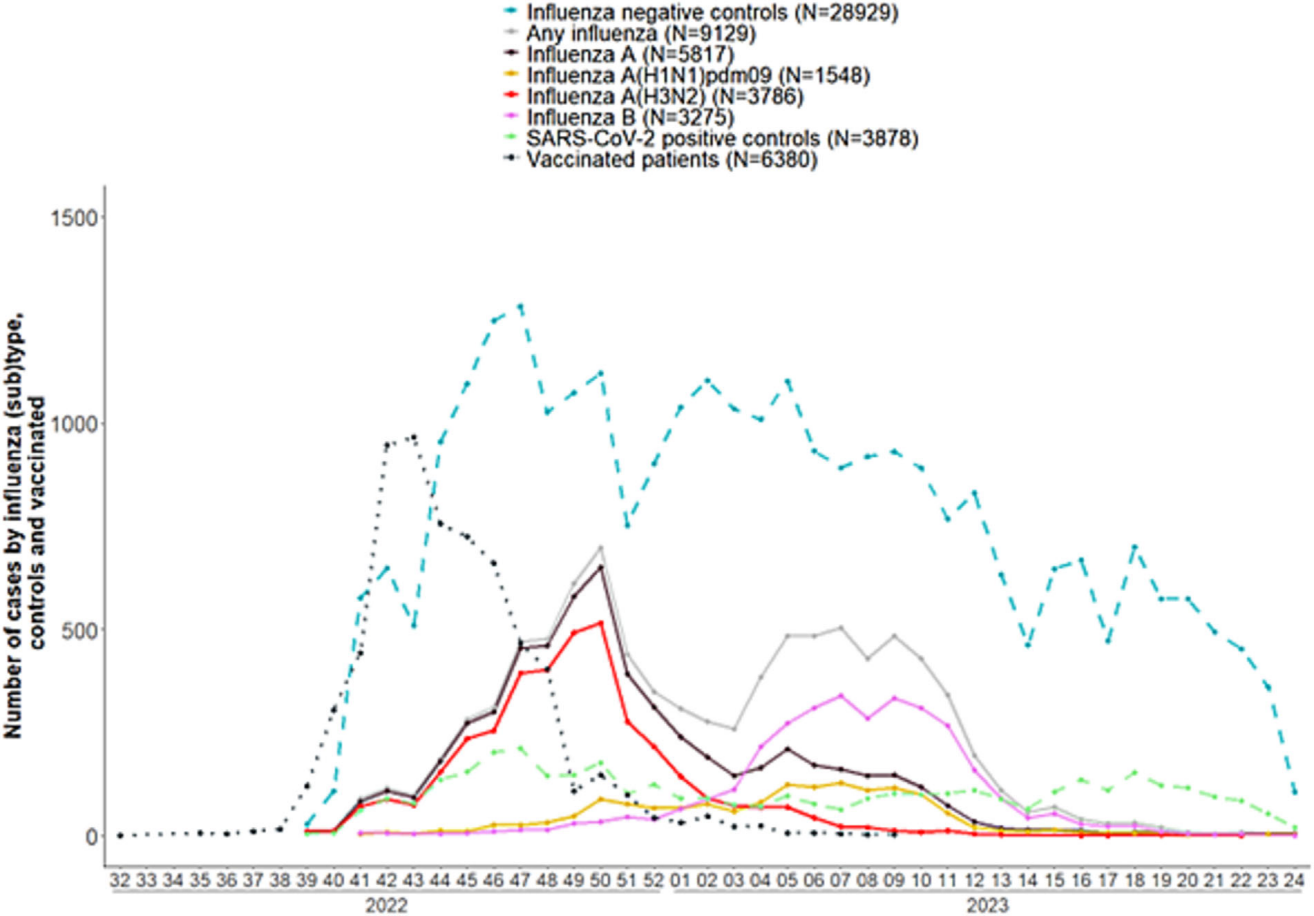
Number of patients by case status and SARS‐CoV‐2 cases among influenza‐negative controls, by week of symptom onset, and numbers of vaccinated patients by week of vaccination, VEBIS primary care multicentre study, Europe, 2022–2023 influenza season.

Overall, the 2022–2023 influenza season across the multicentre study sites was characterised by the co‐circulation of two influenza A subtypes, particularly influenza A(H3N2), and influenza B viruses (Figure 1). We included 9129 influenza‐positive patients with symptom onset from between ISO (International Organization for Standardization) week 39, 2022 up to and including week 24, 2023. Vaccination campaigns were ongoing during the start of influenza circulation, with patients vaccinated up to and including ISO week 9, 2023. There were more SARS‐CoV‐2 positive cases among controls at the beginning of the season, with 30% (*n* = 1175) controls being SARS‐CoV‐2 positive between ISO week 44 and week 50, 2022 (Figure 1).

We included 38,058 patients, of whom 9129 (24%) were influenza positive. Among these, 5,824 were influenza A, 3285 influenza B and 38 influenza not subtyped infections, noting that there were 10 influenza A(H3N2) and B co‐infections, one influenza A(H1N1)pdm09 and B co‐infection and seven influenza A(H3N2) and A(H1N1)pdm09 co‐infections.

Among influenza A infections, 3794 were influenza A(H3N2), 1548 A(H1N1)pdm09 and 482 influenza A cases not subtyped. Because of low sample size, we excluded eight cases from the influenza A(H3N2) analysis from one study site, and 10 cases from the influenza B analysis from three study sites.

There were 28,929 influenza‐negative controls between ISO weeks 39, 2022 and 24, 2023.

The median age was 39 years among controls and 22, 41 and 18 years among influenza A(H3N2) cases, influenza A(H1N1)pdm09 and influenza B cases, respectively. Among controls, 15% were aged 0–4 years compared with 10% and 11% among influenza A(H1N1)pdm09 and A(H3N2) cases, respectively. There were 17% of patients aged 65 and older among controls, 6% among influenza A(H3N2) cases, 9% among influenza A(H1N1)pdm09 cases and 1% among influenza B cases. The proportion of SARS‐CoV‐2 positive controls was 13% (*n* = 3878).

Among controls, the proportion vaccinated with the 2022–2023 influenza vaccine was 20% (*n* = 5767) compared with 7% (*n* = 613) among all influenza cases, 8% (*n* = 288) among influenza A(H3N2) cases, 12% (*n* = 185) among influenza A(H1N1)pdm09 cases and 3% (*n* = 93) among influenza B cases.

Among controls, 17% (*n* = 4396) were part of the target group for influenza vaccination and vaccinated compared with 6% (*n* = 210) among influenza A(H3N2) cases, 11% (*n* = 148) among influenza A(H1N1)pdm09 cases and 16% (*n* = 447) among influenza B cases (Table 1).

**TABLE 1:** Characteristics of all influenza, influenza A, A(H3N2), A(H1N1)pdm09 and B cases, and controls included in the VEBIS primary care multicentre study, Europe, influenza season 2022–2023.

Variable	Test‐negative controls^a^ (*N* = 28,929)	All influenza cases(*N* = 9129)	Influenza A cases(*N* = 5817)	Influenza A(H3N2) cases(*N* = 3786)	Influenza A(H1N1)pdm09 cases(*N* = 1548)	Influenza B cases(*N* = 3275)
Age						
Mean (SD)	37.6 (25.6)	28.1 (20.8)	30.8 (22.3)	27.9 (21.7)	37.7 (22)	23 (16.6)
Median (IQR)	39 (13–58)	25 (10–43)	29 (11–48)	22 (9–45)	41 (17–54)	18 (8–36)
Missing, *n* (%)	15 (0.1)	11 (0.1)	8 (0.1)	6 (0.2)	2 (0.1)	3 (0.1)
Age group (years), *n* (%)						
0–4	4297 (15)	948 (10)	633 (11)	431 (11)	155 (10)	318 (10)
5–14	3312 (11)	2467 (27)	1361 (23)	1048 (28)	198 (13)	1105 (34)
15–64	16,271 (56)	5220 (57)	3383 (58)	2060 (54)	1047 (68)	1804 (55)
≥65	5034 (17)	483 (5)	432 (7)	241 (6)	146 (9)	45 (1)
Missing	15 (0)	11 (0)	8 (0)	6 (0)	2 (0)	3 (0)
Sex, *n* (%)						
Female	16,574 (57)	4659 (51)	3012 (52)	1953 (52)	818 (53)	1620 (50)
Male	12,305 (43)	4447 (49)	2789 (48)	1819 (48)	728 (47)	1648 (50)
Missing	50 (0)	23 (0)	16 (0)	14 (0)	2 (0)	7 (0)
Seasonal influenza vaccination, *n* (%)						
No	23,162 (80)	8516 (93)	5298 (91)	3498 (92)	1363 (88)	3182 (97)
Yes	5767 (20)	613 (7)	519 (9)	288 (8)	185 (12)	93 (3)
Missing	0 (0)	0 (0)	0 (0)	0 (0)	0 (0)	0 (0)
Delay between influenza vaccination and onset of symptoms in days						
Mean (SD)	100.6 (57.5)	75.1 (41.7)	69.6 (39.2)	55.9 (30.5)	90.9 (40.5)	106 (42.3)
Median (IQR)	92 (53–144)	67 (43–103)	63 (39–93)	52 (32.5–70.2)	89 (61–120)	105 (75–128)
Delay between onset of symptoms and swabbing in days						
Mean (SD)	2.6 (1.8)	2.5 (1.6)	2.4 (1.6)	2.4 (1.6)	2.3 (1.6)	2.6 (1.6)
Median (IQR)	2 (1–4)	2 (1–3)	2 (1–3)	2 (1–3)	2 (1–3)	2 (1–4)
Missing, *n* (%)	0 (0)	0 (0)	0 (0)	0 (0)	0 (0)	0 (0)
Any chronic condition, *n* (%)						
No presence of chronic disease	17,724 (71)	6764 (84)	4233 (81)	2917 (84)	1047 (76)	2521 (89)
Presence of chronic disease	7409 (29)	1292 (16)	978 (19)	570 (16)	332 (24)	311 (11)
Missing	3796 (13)	1073 (12)	606 (10)	299 (8)	169 (11)	443 (14)
Target group for influenza vaccination^b^, *n* (%)						
No	14,741 (58)	6050 (75)	3717 (71)	2591 (74)	876 (64)	2324 (82)
Yes	10,793 (42)	2003 (25)	1501 (29)	890 (26)	497 (36)	496 (18)
Missing	3395 (12)	1076 (12)	599 (10)	305 (8)	175 (11)	455 (14)
Target group for influenza vaccination^b^ by vaccination status, *n* (%)						
No; vaccinated	925 (4)	134 (2)	101 (2)	66 (2)	26 (2)	34 (1)
No; unvaccinated	13,816 (54)	5916 (73)	3616 (69)	2525 (73)	850 (62)	2290 (81)
Yes; vaccinated	4396 (17)	440 (5)	389 (7)	210 (6)	148 (11)	447 (16)
Yes; unvaccinated	6397 (25)	1563 (19)	1112 (21)	680 (20)	349 (25)	49 (2)
Missing; vaccinated	446 (2)	39 (0)	29 (0)	12 (0)	11 (1)	10 (0)
Missing; unvaccinated	2949 (10)	1037 (11)	570 (10)	293 (8)	164 (11)	445 (14)
SARS‐CoV‐2 PCR test result, *n* (%)						
Negative	25,033 (87)	8967 (98)	5707 (98)	3728 (99)	1514 (98)	3226 (99)
Positive	3878 (13)	138 (2)	97 (2)	55 (1)	30 (2)	38 (1)
Missing	18 (0)	24 (0)	13 (0)	3 (0)	4 (0)	11 (0)

Abbreviation: VEBIS, Vaccine Effectiveness, Burden and Impact Studies

^a^ Controls for ‘any influenza’ used here (number of controls differs slightly for influenza A(H1N1)pdm09 and B analyses, due to the inclusion criteria).

^b^ Target group, varying among countries, included patients with chronic conditions, pregnant women (for some countries), older adults (those aged 50, 60 or 65 and older depending on study site), patients belonging to other risk groups (e.g. health care workers and other professional groups). In one study site, it included children aged 2–17 years of age.

### 3.1 Genetic characterisation

Thirty‐four percent (1225/3566) influenza A(H3N2), 29% (402/1374) influenza A(H1N1)pdm09 and 18% (550/3098) influenza B samples were sequenced in the nine study sites and laboratories performing sequencing.

Among the 1225 influenza A(H3N2) sequenced samples, 281 (23%) belonged to clade 2a.1b, 4% (*n* = 52) to clade 2a.1, 9% (*n* = 112) to clade 2a.3 and 64% (*n* = 780) to clade 2b (Table 2). Regardless of clade, 12% (*n* = 143) of sequenced viruses had the T135A mutation.

**TABLE 2:** Influenza viruses characterised by clade, amino acid substitutions, VEBIS primary care multicentre study, Europe, influenza season 2022–2023.

Characterised viruses	Clade	*N* (%)
Influenza A(H3N2) (*n* = 1225)		
	2a.1	52 (4)
	2a.1b	281 (23)
	2a.3	112 (9)
	2b	780 (64)
Influenza A(H1N1)pdm09 (*n* = 402)		
	5a.2a.1	77 (19)
	5a.2a	322 (80)
	5a.1	3 (1)
Influenza B Victoria (*n* = 550)		
With D197E mutation		323 (59)
	Cluster (i) T182A, T221A	42
	Cluster (iv) D129G/N	13
	Cluster (vi) E183K	102
	Cluster V117I	70
	V1A.3a.2^a^	96
Without D197E mutation		227 (41)
	Cluster (ii) E128K	162
	Cluster (iii) E198G	65

Abbreviation: VEBIS, Vaccine Effectiveness, Burden and Impact Studies

^a^ V1A.3a.2 with no changes or mutations listed above.

Among the 402 influenza A(H1N1)pdm09 sequenced samples, 322 (80%) belonged to clade 5a.2a, 77 (19%) belonged to clade 5a.2a.1 and 3 (1%) belonged to clade 5a.1.

Among the 550 influenza B sequenced samples, 323 (59%) belonged to clusters harbouring the D197E mutation and 227 (41%) belonged to clusters without the D197E mutation.

### 3.2 Vaccine effectiveness estimates

We excluded 13% of patients from the complete case analysis against any influenza, influenza A, A(H3N2), A(H1N1)pdm09 and 14% from the complete case analysis against influenza B, because of missing values for sex (0.2–0.3%), age (0.1%) and chronic condition (13–14%), depending on the analysis.

#### 3.2.1 Any influenza

**FIGURE 2:**
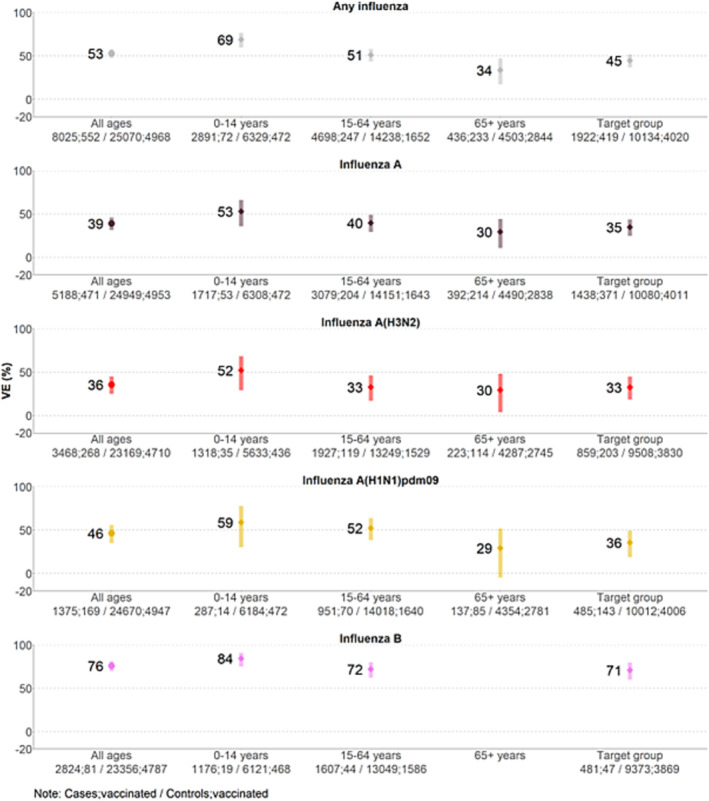
Pooled seasonal vaccine effectiveness against any influenza, influenza A, A(H3N2), A(H1N1)pdm09 and B overall, by age group and among the influenza vaccination target group. VEBIS primary care multicentre study, Europe, influenza season 2022–2023.

The overall VE against infection with any influenza was 53% (95% CI: 48–58) for all ages combined. The VE was 69% (95% CI: 60–76) among 0–14‐year‐olds, 51% (95% CI: 44–58) among 15–64‐year‐olds and 34% (95% CI: 17–47) among those aged 65 years and older. The VE among patients in the influenza vaccination target group was 45% (95% CI: 37–52) (Figure 2; Table S1).

#### 3.2.2 Influenza A

Against influenza A, overall VE was 39% (95% CI: 31–46). The VE was 53% (95% CI: 36–66) among 0–14‐year‐olds, 40% (95% CI: 29–49) among 15–64‐year‐olds and 30% (95% CI: 11–44) among those aged ≥65 years. Among those in the target group for influenza vaccination, VE was 35% (95% CI: 25–44) (Figure 2; Table S1).

#### 3.2.3 Influenza A(H3N2)

Against influenza A(H3N2), overall VE was 36% (95% CI: 25–45). The VE was 52% (95% CI: 29–68) among 0–14‐year‐olds, 33% (95% CI: 17–46) among 15–64‐year‐olds and 30% (95% CI: 4–48) among those aged ≥65 years. Among those in the target group for influenza vaccination, VE was 33% (95% CI: 18–45) (Figure 2; Table S1).

The all‐age VE against influenza A(H3N2) was 45% (95% CI: 34–55) among those presenting 14–59 days, 11% (95% CI: −15–32) among those presenting 60–89 days and 7% (95% CI: −38–40) among those presenting ≥90 days since vaccination (Table 3).

**TABLE 3:** Pooled seasonal vaccine effectiveness against influenza a(H3N2), a(H1N1)pdm09 and B, overall, by time since vaccination, VEBIS primary care multicentre study, Europe, influenza season 2022–2023.

Influenza type/subtype	Study population	Time since vaccination (days)	*N* ^a^	Cases	Cases vaccinated	Controls	Controls vaccinated	VE (95% CI)
Influenza A(H3N2)	All ages	14–59	23,342	3360	160	19,982	1523	45 (34–55)
		60–89	22,666	3280	80	19,386	927	11 (‐15–32)
		≥90	23,947	3228	28	20,719	2260	7 (‐38–40)
Influenza A(H1N1)pdm09	All ages	14–89	23,513	1294	88	22,219	2496	49 (35–60)
		90–119	21,724	1244	38	20,480	757	47 (25–64)
		≥120	22,666	1249	43	21,417	1694	24 (‐7–47)
Influenza B	All ages	14–89	23,688	2772	29	20,916	2347	80 (71–86)
		90–119	22,082	2766	23	19,316	747	78 (67–86)
		≥120	23,034	2772	29	20,262	1693	66 (50–78)

Abbreviations: VEBIS, Vaccine Effectiveness, Burden and Impact Studies; *N*, number; VE, vaccine effectiveness; CI, confidence interval.

^a^ Based on the complete case analysis: records with missing age, sex and chronic condition are dropped.

The overall VE against clade 2a.1 or 2a.1b was 38% (95% CI: 19–52), 38% (95% CI: 25–49) against clade 2b and 72% (95% CI: 47–88) against A(H3N2) with a mutation at position T135A (Table 4).

**TABLE 4:** Pooled seasonal vaccine effectiveness against clades and genetic variants of influenza a(H3N2), a(H1N1)pdm09 and B overall, VEBIS primary care multicentre study, Europe, influenza season 2022–2023.

Influenza type/subtype	Study population	Clade	*N* ^a^	Cases	Cases vaccinated	Controls	Controls vaccinated	VE (95% CI)
Influenza A(H3N2)	All ages	2a.1 or 2a.1b	13,953	324	27	13,629	2630	38 (19–52)
		2a.1b	13,610	269	26	13,341	2564	24 (1–43)
		2b	16,721	767	48	15,954	3209	38 (25–49)
		T135A mutation	11,042	133	3	10,909	1893	72 (47–88)
Influenza A(H1N1)pdm09	All ages	5a.2a	17,629	308	43	17,321	3935	56 (46–65)
		5a.2a.1	12,277	70	6	12,207	2558	79 (64–88)
Influenza B	All ages	D197E mutation	16,647	309	10	16,338	3562	79 (73–85)
		No D197E mutation	13,207	220	5	12,987	3027	90 (85–94)

Abbreviations: VEBIS, Vaccine Effectiveness, Burden and Impact Studies; *N*, number; VE, vaccine effectiveness; CI, confidence interval.

^a^ Based on the complete case analysis: records with missing age, sex and chronic condition are dropped.

#### 3.2.4 Influenza A(H1N1)pdm09

The VE against influenza A(H1N1)pdm09 was 46% (95% CI: 35–56) overall, 59% (95% CI: 30–78) among 0–14‐year‐olds, 52% (95% CI: 38–64) among 15–64‐year‐olds and 29% (95% CI: −4–52) among those aged ≥65 years. Among those in the influenza vaccination target group, VE was 36% (95% CI: 19–49) (Figure 2; Table S1).

The VE against influenza A(H1N1)pdm09 (all ages) was 49% (95% CI: 35–60) among those presenting 14–89 days, 47% (95% CI: 25–64) among those presenting 90–119 days and 24% (95% CI: −7–47) among those presenting ≥120 days since vaccination (Table 3).

Among all ages, overall VE against clade 5a.2a was 56% (95% CI: 46–65) and 79% (95% CI: 64–88) against clade 5a.2a.1 (Table 4).

#### 3.2.5 Influenza B

Against influenza B, overall VE was 76% (95% CI: 70–81), 84% (95% CI: 75–91) among 0–14‐year‐olds, and 72% (95% CI: 62–80) among 15–64‐year‐olds. Sample size did not permit estimation of VE among those aged 65 years and older. Among those in the target group for influenza vaccination, VE was 71% (95% CI: 60–80) (Figure 2; Table S1).

The VE against influenza B (all ages) was 80% (95% CI: 71–86) among those presenting 14–89 days, 78% (95% CI: 67–86) among those presenting 90–119 days and 66% (95% CI: 50–78) among those presenting ≥120 days since vaccination (Table 3).

The overall VE against influenza B with a mutation at position 197 was 79% (95% CI: 73–85); influenza B VE was 90% (95% CI: 85–94) without this mutation position (Table 4).

#### 3.2.6 Sensitivity analysis—excluding SARS‐CoV‐2 positive controls

In this analysis, VE point estimates differed by <4% absolute for all influenza (sub)types and age groups (Table S2).

## 4 DISCUSSION

Influenza A(H1N1)pdm09, A(H3N2) and B circulated in the VEBIS primary care multicentre study during the 2022–2023 influenza season. The VE against any influenza among all ages was 53%, ranging between 34% and 69% by age group and was 45% among those in the target group for influenza vaccination. Against influenza A(H3N2), VE was 36% among all ages, ranging between 30% and 52% by age and target groups. The VE against influenza A(H1N1)pdm09 was 46% among all ages and the VE ranged between 29% and 59% by age and target groups. The VE against influenza B was 76% overall, ranging from 72% to 84% in those <65 years old or in an influenza vaccine target group (sample size was too low to estimate VE for those ≥65 years old). Against all influenza (sub)types, VE point estimates were higher among those aged 0–14 years than among those aged 15–64 years or 65 years and older.

The 2022–2023 VEBIS primary care multicentre study end‐of‐season VE estimate against any influenza among all ages was higher at 53% compared to the interim 2022–2023 estimate of 44%.^16^ This higher VE is particularly due to the late circulation of influenza B, which had a high VE. The 2022–2023 VEBIS primary care multicentre study end‐of‐season VE estimate against influenza A among all ages was similar to the interim 2022–2023 estimate (39% vs 40%, respectively).

The all‐age VE estimates against influenza A(H3N2) were higher at 37% than estimates in all past seasons since 2013–2014 within the I‐MOVE primary care multicentre studies, with the exception of the 2019–2020 season, where the VE was 49%.^17^ The VE was lower than the 2022–2023 interim VE estimates from Canada (47%–59%) and that from Wisconsin in the United States (60%).^18^, ^19^ The majority of the viruses sequenced (64%) belonged to the 2b clade, which is characterised by the mutations E50K, F79V, I140K and S156H compared with the vaccine virus. The VE was 38% against the 2b clade and clades 2a.1 and 2a.1b combined. The VE against A(H3N2) with the T135A substitution—in the antigenic site A in the influenza haemagglutinin—was 72%. In 2018–2019 within our network, we observed lower VE among viruses with substitutions at the T135K position.^18^ This mutation results in the loss of a glycosylation site, which is associated with antigenic change, and we would expect a lower VE. Researchers in Canada observed a slightly lower VE among patients with A(H3N2) viruses with a T135K substitution, also resulting in a loss of glycosylation, compared to those without this substitution, but point estimates differed by <10%.^18^ Within our study, among patients with sequenced viruses, 62% (89/143) of patients with the T135A substitution were aged 0–14 years, compared with 41% (438/1079) of patients without the T135A substitution (data not shown). Overall, VE against A(H3N2) was higher among children, although sample size was too small to disentangle age and virological effects, that is to determine if there were differences in VE against influenza A(H3N2) among children with and without this mutation. In general, antiserum raised against the egg‐propagated vaccine virus recognised circulating A(H3N2) viruses well.^20^ For influenza A(H3N2), the VE was 45% among those vaccinated 14–59 days before symptom onset and 7% among those vaccinated 90 days or more before symptom onset. Waning of the vaccine effect against influenza A(H3N2) has been observed in previous seasons, but it appears to have been more rapid this season.^8^, ^21^

We observed VE estimates against influenza A(H1N1)pdm09 similar to previous seasons, often at around 50% overall across seasons since the 2009 A(H1N1)pdm09 pandemic.^7^, ^8^, ^22^ An exception was in the 2021–2022 season where the VE against A(H1N1)pdm09 was higher at 75% and in the 2015–2016 season where VE against A(H1N1)pdm09 was lower at 33%.^7^, ^8^ Influenza A(H1N1)pdm09 VE point estimates in the 2022–2023 season were lower at 29% among those aged 65 years and older, compared with other age groups, but confidence intervals overlapped. Among the sequenced viruses, 80% belonged to the 5a.2a clade, represented by A/Sydney/5/2021, harbouring K54Q, A186T, Q189E, E224A, R259K and K308R substitutions compared with the vaccine strain. Most others belonged to 5a.2a.1, represented by A/Norway/25089/2022, harbouring additionally the P137S, K142R and D260E substitutions compared with the vaccine virus. (Sub)clade‐specific VE was 56% and 79% against clade 5a.2a and clade 5a.2a.1, respectively, with overlapping CI, indicating no differences between circulating strains. These results do not support antigenic results, where human post‐vaccination sera poorly recognised 5a.2a.1 viruses.^23^

The VE point estimate was 47%–49% among those vaccinated 14–119 days before symptom onset and 24% among those vaccinated 120 days or more before symptom onset. There has been little evidence in the past within this network for a decline in VE against A(H1N1)pdm09 within the season.^18^ The precision around the estimates does not allow inference as to whether there is a true decline in VE, and (sub)clade‐specific VE does not indicate any decline in VE due to virological changes during the season. Although the lower point estimate among those with 120 or more days since vaccination may be a chance finding, we will repeat analyses in subsequent seasons to help understand any potential waning immunity against the A(H1N1)pdm09 vaccine.

The 2022–2023 influenza B viruses showed the distinct B/Victoria lineage age pattern of a higher proportion of cases aged <15 and 20–40 years. There were few influenza B cases (1%) among those aged 65 years and over. There was some evidence of decline in VE against influenza B, from 80% among those vaccinated 14–89 days, to 66% among those vaccinated 120 days or more before symptom onset, but confidence intervals overlapped. VE against influenza B was high with all point estimates ≥66%. Circulating viruses were antigenically similar to the cell culture‐ or egg‐propagated B/Austria/1359417/2021 vaccine virus.^23^

In the primary analysis, we included SARS‐CoV‐2 positive controls. Excluding these did not change the VE estimates by much more than 3%, indicating that there is unlikely to be bias relating to the correlation between influenza and COVID‐19 vaccination in this setting. This could be due to the low number of SARS‐CoV‐2 positive controls (13%, *n* = 3878), or a low COVID‐19 VE in an outpatient VE setting, with a long study period and thus considerable time since vaccination, or both.

This season our study reached its highest sample size since the primary care network started estimating influenza VE, as I‐MOVE, in the 2008–2009 season.^3^ However, one of the study sites (Spain, national level) represented 67% of the data, thus having more weight in the analysis than other study sites. Additional limitations included the low number of influenza B cases among older adults, which resulted in an inability to estimate the VE against influenza B in this age group. We could not estimate VE by vaccine type/brand, as not all study sites could provide this information in the 2022–2023 season. VE estimates by clade were higher than the general ones for influenza A(H3N2) and A(H1N1); this may be due to random variation relating to the smaller sample size among sequenced viruses. As with all observational studies, our study could be subject to bias because of unmeasured confounding. In the context of a post‐COVID‐19 pandemic era, changes in behaviour (including healthcare‐seeking behaviour) of the source population in this multicentre study may affect our estimates. However, the use of the test‐negative design, with the selection of patients according to a designated clinical picture, may help alleviate some biases relating to this. Also, our results are similar to those of other studies, in the current season and past seasons since the COVID‐19 pandemic. These findings increase confidence in the interpretation of our results.

## 5 CONCLUSION

The 2022–2023 end‐of‐season results from the VEBIS network at primary care level showed high VE against influenza B, with lower VE against influenza A(H1N1)pdm09 and A(H3N2). The VE was higher among children, with point estimates exceeding 50% for all influenza (sub)types. Overall, VE indicated that around one in two vaccinated people were protected against influenza presentation at primary care level in our study.

## AUTHOR CONTRIBUTIONS

**Marine Maurel:** Conceptualization; data curation; formal analysis; methodology; software; validation; visualization; writing—original draft; writing—review and editing. **Francisco Pozo:** Data curation; formal analysis; investigation; methodology; resources; writing—review and editing. **Gloria Pérez‐Gimeno:** Data curation; formal analysis; investigation; methodology; resources; writing—review and editing. **Silke Buda:** Data curation; formal analysis; investigation; methodology; resources; writing—review and editing. **Noémie Sève:** Data curation; formal analysis; investigation; methodology; resources; writing—review and editing. **Beatrix Oroszi:** Data curation; formal analysis; investigation; methodology; resources; writing—review and editing. **Mariette Hooiveld:** Data curation; formal analysis; investigation; methodology; resources; writing—review and editing. **Verónica Gómez:** Data curation; formal analysis; investigation; methodology; resources; writing—review and editing. **Lisa Domegan:** Data curation; formal analysis; investigation; methodology; resources; writing—review and editing. **Iván Martínez‐Baz:** Data curation; formal analysis; investigation; methodology; resources; writing—review and editing. **Maja Ilić:** Data curation; formal analysis; investigation; methodology; resources; writing—review and editing. **AnnaSara Carnahan:** Data curation; formal analysis; investigation; methodology; resources; writing—review and editing. **Maria Elena Mihai:** Data curation; formal analysis; investigation; methodology; resources; writing—review and editing. **Ana Martinez:** Data curation; formal analysis; investigation; methodology; resources; writing—review and editing. **Luise Goerlitz:** Data curation; formal analysis; investigation; methodology; resources; writing—review and editing. **Vincent ENOUF:** Data curation; formal analysis; investigation; methodology; resources; writing—review and editing. **Judit Krisztina Horvath:** Data curation; formal analysis; investigation; methodology; resources; writing—review and editing. **Frederika Dijkstra:** Data curation; formal analysis; investigation; methodology; resources; writing—review and editing. **Ana Paula Rodrigues:** Data curation; formal analysis; investigation; methodology; resources; writing—review and editing. **Charlene Bennett:** Data curation; formal analysis; investigation; methodology; resources; writing—review and editing. **Camino Trobajo‐Sanmartín:** Data curation; formal analysis; investigation; methodology; resources; writing—review and editing. **Ivan Mlinarić:** Data curation; formal analysis; investigation; methodology; resources; writing—review and editing. **Neus Latorre‐Margalef:** Data curation; formal analysis; investigation; methodology; resources; writing—review and editing. **Alina Ivanciuc:** Data curation; formal analysis; investigation; methodology; resources; writing—review and editing. **Aurora Lopez:** Data curation; formal analysis; investigation; methodology; resources; writing—review and editing. **Ralf Dürrwald:** Data curation; formal analysis; investigation; methodology; resources; writing—review and editing. **Alessandra Falchi:** Data curation; formal analysis; investigation; methodology; resources; writing—review and editing. **Gergő Túri:** Data curation; formal analysis; investigation; methodology; resources; writing—review and editing. **Adam Meijer:** Data curation; formal analysis; investigation; methodology; resources; writing—review and editing. **Aryse Melo:** Data curation; formal analysis; investigation; methodology; resources; writing—review and editing. **Joan O'Donnell:** Data curation; formal analysis; investigation; methodology; resources; writing—review and editing. **Jesus Castilla:** Data curation; formal analysis; investigation; methodology; resources; writing—review and editing. **Vesna Višekruna Vučina:** Data curation; formal analysis; investigation; methodology; resources; writing—review and editing. **Tove Samuelsson Hagey:** Data curation; formal analysis; investigation; methodology; resources; writing—review and editing. **Mihaela Lazar:** Data curation; formal analysis; investigation; methodology; resources; writing—review and editing. **Marlena Kaczmarek:** Conceptualization; visualization; writing—review and editing. **Sabrina Bacci:** Conceptualization; visualization; writing—review and editing. **Esther Kissling:** Conceptualization; formal analysis; funding acquisition; methodology; project administration; software; supervision; validation; visualization; writing—review and editing. **VEBIS study team:** Data curation; formal analysis; investigation; methodology; resources; writing—review and editing.

## CONFLICT OF INTEREST STATEMENT

None.

### PEER REVIEW

The peer review history for this article is available at https://www.webofscience.com/api/gateway/wos/peer-review/10.1111/irv.13243.

## ETHICS STATEMENT

The planning, conduct and reporting of the studies were in line with the Declaration of Helsinki. Some studies did not require official ethical approval or patient consent as they are part of routine care/surveillance: Spain. In the Netherlands, as the data are initially collected through surveillance, no formal ethical approval was necessary. Verbal informed consent from patients for participation in the national respiratory surveillance is required. In addition, patients have the option to opt out for participation in any further research (including VE studies). Other study sites received local ethical approval from a national or regional review board: Croatia: approved by the Ethics Committee of the Croatian Institute of Public Health (class 030‐02/22‐01/4 and class 030‐02/23‐01/1); France: 471393; Germany: EA2/126/11; Hungary: approved by the National Scientific and Ethical Committee (IV/1885‐5/2021/EKU); Ireland: ICGP2019.4.04; Portugal: approved 18 January 2012 by the Ethics Committee of Instituto Nacional de Saúde Doutor Ricardo Jorge, no registration number given; Romania: CE199/2022; Sweden: 2006/1040‐31/2.

## Supporting information


**Table S1:** Pooled seasonal vaccine effectiveness against any influenza, influenza A, A(H3N2), A(H1N1)pdm09 and B, overall, by age group and among target group. VEBIS primary care multicentre study, Europe, influenza season 2022–23.
**Table S2:** Pooled seasonal vaccine effectiveness against any influenza, influenza A, A(H3N2), A(H1N1)pdm09 and B, overall, by age group and among target group excluding SARS‐CoV‐2 positive controls. VEBIS primary care multicentre study, Europe, influenza season 2022–23.

## Data Availability

Data are available only on request due to privacy/ethical restrictions: The data that support the findings of this study may be available on request from the corresponding author, depending on the request. The data are not publicly available due to privacy or ethical restrictions.
