# Supplementary material for: Influenza vaccine effectiveness in Europe: Results from the 2022–2023 VEBIS (Vaccine Effectiveness, Burden and Impact Studies) primary care multicentre study
Source: Influenza Other Respir Viruses. 2024 Jan 10;18(1):e13243. doi: 10.1111/irv.13243 (PMC10777262; doi:10.1111/irv.13243)
Supplement: Supplementary file 1 — Table S1: Pooled seasonal vaccine effectiveness against any influenza, influenza A, A(H3N2), A(H1N1)pdm09 and B, overall, by age group and among target group. VEBIS primary care multicentre study, Europe, influenza season 2022–23. Table S2: Pooled seasonal vaccine effectiveness against any influenza, influenza A, A(H3N2), A(H1N1)pdm09 and B, overall, by age group and among target group excluding SARS‐CoV‐2 positive controls. VEBIS primary care multicentre study, Europe, influenza season 2022–23. [file IRV-18-e13243-s001.docx]

**Supplementary material**

**Table S1:** Pooled seasonal vaccine effectiveness against any influenza, influenza A, A(H3N2), A(H1N1)pdm09 and B, overall, by age group and among target group. VEBIS primary care multicentre study, Europe, influenza season 2022–23.

| **Influenza type/subtype** | **Study population** | **N**^†^ | **Cases** | **Cases vaccinated** | **Controls** | **Controls vaccinated** | **VE (95% CI)** |
| --- | --- | --- | --- | --- | --- | --- | --- |
| Any influenza | All ages | 33 095 | 8 025 | 552 | 25 070 | 4 968 | 53 (48–58) |
|  | 0-14 years | 9 220 | 2 891 | 72 | 6 329 | 472 | 69 (60–76) |
|  | 15-64 years | 18 936 | 4 698 | 247 | 14 238 | 1 652 | 51 (44–58) |
|  | 65+ years | 4 939 | 436 | 233 | 4 503 | 2 844 | 34 (17–47) |
|  | Target group | 12 056 | 1 922 | 419 | 10 134 | 4 020 | 45 (37–52) |
| Influenza A | All ages | 30 137 | 5 188 | 471 | 24 949 | 4 953 | 39 (31–46) |
|  | 0-14 years | 8 025 | 1 717 | 53 | 6 308 | 472 | 53 (36–66) |
|  | 15-64 years | 17 230 | 3 079 | 204 | 14 151 | 1 643 | 40 (29–49) |
|  | 65+ years | 4 882 | 392 | 214 | 4 490 | 2 838 | 30 (11–44) |
|  | Target group | 11 518 | 1 438 | 371 | 10 080 | 4 011 | 35 (25–44) |
| Influenza A(H3N2) | All ages | 26 637 | 3 468 | 268 | 23 169 | 4 710 | 36 (25–45) |
|  | 0-14 years | 6 951 | 1 318 | 35 | 5 633 | 436 | 52 (29–68) |
|  | 15-64 years | 15 176 | 1 927 | 119 | 13 249 | 1 529 | 33 (17–46) |
|  | 65+ years | 4 510 | 223 | 114 | 4 287 | 2 745 | 30 (4–48) |
|  | Target group | 10 367 | 859 | 203 | 9 508 | 3 830 | 33 (18–45) |
| Influenza  A(H1N1)pdm09 | All ages | 26 045 | 1 375 | 169 | 24 670 | 4 947 | 46 (35–56) |
|  | 0-14 years | 6 471 | 287 | 14 | 6 184 | 472 | 59 (30–78) |
|  | 15-64 years | 14 969 | 951 | 70 | 14 018 | 1 640 | 52 (38–64) |
|  | 65+ years | 4 491 | 137 | 85 | 4 354 | 2 781 | 29 (-4–52) |
|  | Target group | 10 497 | 485 | 143 | 10 012 | 4 006 | 36 (19–49) |
| Influenza B | All ages | 26 180 | 2 824 | 81 | 23 356 | 4 787 | 76 (70–81) |
|  | 0-14 years | 7 297 | 1 176 | 19 | 6 121 | 468 | 84 (75–91) |
|  | 15-64 years | 14 656 | 1 607 | 44 | 13 049 | 1 586 | 72 (62–80) |
|  | Target group | 9 854 | 481 | 47 | 9 373 | 3 869 | 71 (60–80) |
| † Based on the complete case analysis: records with missing age, sex and chronic condition are dropped.  Abbreviations: VEBIS, Vaccine Effectiveness, Burden and Impact Studies; N, number; VE, vaccine effectiveness; CI, confidence interval | | | | | | | |

**Table S2:** Pooled seasonal vaccine effectiveness against any influenza, influenza A, A(H3N2), A(H1N1)pdm09 and B, overall, by age group and among target group excluding SARS-CoV-2 positive controls. VEBIS primary care multicentre study, Europe, influenza season 2022–23.

| **Influenza type/subtype** | **Study population** | **N**^†^ | **Cases** | **Cases vaccinated** | **Controls** | **Controls vaccinated** | **VE (95% CI)** |
| --- | --- | --- | --- | --- | --- | --- | --- |
| Any influenza | All ages | 29 732 | 8 025 | 552 | 21 707 | 4 285 | 54 (49–59) |
|  | 0-14 years | 8 936 | 2 891 | 72 | 6 045 | 456 | 70 (60–77) |
|  | 15-64 years | 16 599 | 4 698 | 247 | 11 901 | 1 394 | 52 (44–58) |
|  | 65+ years | 4 197 | 436 | 233 | 3 761 | 2 435 | 36 (20–49) |
|  | Target group | 10 464 | 1 922 | 419 | 8 542 | 3 445 | 46 (38–53) |
| Influenza A | All ages | 26 798 | 5 188 | 471 | 21 610 | 4 275 | 41 (33–48) |
|  | 0-14 years | 7 743 | 1 717 | 53 | 6 026 | 456 | 54 (38–67) |
|  | 15-64 years | 14 910 | 3 079 | 204 | 11 831 | 1 388 | 41 (30–50) |
|  | 65+ years | 4 145 | 392 | 214 | 3 753 | 2 431 | 32 (14–46) |
|  | Target group | 9 936 | 1 438 | 371 | 8 498 | 3 439 | 36 (26–45) |
| Influenza A(H3N2) | All ages | 23 554 | 3 468 | 268 | 20 086 | 4 083 | 39 (28–48) |
|  | 0-14 years | 6 697 | 1 318 | 35 | 5 379 | 422 | 54 (31–70) |
|  | 15-64 years | 13 019 | 1 927 | 119 | 11 092 | 1 292 | 34 (19–48) |
|  | 65+ years | 3 838 | 223 | 114 | 3 615 | 2 369 | 32 (8–50) |
|  | Target group | 8 905 | 859 | 203 | 8 046 | 3 299 | 34 (20–46) |
| Influenza A(H1N1)pdm09 | All ages | 22 733 | 1 375 | 169 | 21 358 | 4 270 | 47 (36–56) |
|  | 0-14 years | 6 193 | 287 | 14 | 5 906 | 456 | 60 (31–78) |
|  | 15-64 years | 12 668 | 951 | 70 | 11 717 | 1 385 | 52 (38–64) |
|  | 65+ years | 3 782 | 137 | 85 | 3 645 | 2 386 | 32 (-1–54) |
|  | Target group | 8 925 | 485 | 143 | 8 440 | 3 435 | 37 (20–50) |
| Influenza B | All ages | 23 128 | 2 824 | 81 | 20 304 | 4 150 | 76 (70–81) |
|  | 0-14 years | 7 020 | 1 176 | 19 | 5 844 | 452 | 84 (75–91) |
|  | 15-64 years | 12 552 | 1 607 | 44 | 10 945 | 1 342 | 72 (61–80) |
|  | Target group | 8 418 | 481 | 47 | 7 937 | 3 333 | 69 (57–78) |
| † Based on the complete case analysis: records with missing age, sex and chronic condition are dropped.  Abbreviations: VEBIS, Vaccine Effectiveness, Burden and Impact Studies; N, number; VE, vaccine effectiveness; CI, confidence interval | | | | | | | |
